# Charged Topics in Medical Education: Students’ Perspectives on Power, Voices, and Faculty Engagement

**DOI:** 10.5334/pme.2253

**Published:** 2026-02-03

**Authors:** Maram Alkhatib, Shazia Samanani, Megan Murphy, Bridget O’Brien, Zareen Zaidi

**Affiliations:** 1 Wake Forest School of Medicine, Charlotte, North Carolina, USA; 2 George Washington University School of Medicine and Health Sciences, Washington, District of Columbia, USA; 3 George Washington University Medical Faculty Associates Internal Medicine Department, Washington, District of Columbia, USA; 4 University of California, San Francisco, San, Francisco, California, USA

## Abstract

**Introduction:**

Medical education learning environments involve charged topics at the intersection of social, political, and cultural domains. Growing student diversity and political polarization add complexity to the discussion of charged topics, yet current literature lacks insight into student perspectives. This study explores how medical students position themselves during charged topic discussions and their preferences for educators’ engagement.

**Methods:**

Using educational cultural hegemony as a theoretical framework and a critical constructivist approach, the research team conducted semi-structured interviews with sensitizing scenarios addressing three charged topics: gun control, reproductive healthcare coverage, and affirmative action. Following IRB approval, twenty medical students across all training years were interviewed. Transcripts were analyzed using Braun and Clarke’s reflexive thematic analysis.

**Findings:**

We identified four themes: ‘Constrained Voices’ describes how power hierarchies influence student engagement with charged conversations. ‘Burdened Voices’ reveals how marginalized students bear a disproportionate burden when educator avoidance of discussions can reinforce dominant perspectives. ‘Finding Voice’ highlights how students use personal experience to center patient perspectives despite hierarchical constraints. In ‘Guiding Voice’, students describe the ideal educator as one who uses their experience and evidence-based knowledge to facilitate discussions.

**Conclusion:**

Power hierarchies constrain student engagement in charged discussions, with marginalized students bearing a disproportionate burden when educators avoid these topics. Despite these constraints, students assert agency through personal experience to center patient perspectives. Students seek educators who actively guide discussions using expertise and evidence, viewing neutrality as itself a stance. The findings underscore the need for faculty development addressing these dynamics.

## Introduction

Medical education culture often discourages open discussion of sensitive topics, including politics, religion, and personal values, despite their influence on patient care [1,2]. Yet diverse classrooms offer rich opportunities to discuss such topics in ways that support the development of cultural humility and understanding of how cultural values shape health beliefs and treatment preferences [3,4].

At the same time, many topics in higher education in general feel difficult or unsafe for students and faculty to discuss openly. Recent surveys showed that nearly half of college students choose not to share what might be considered unpopular political opinions, and many educators self-censor in professional settings [5,6]. With up to 70% of students believing professors should be reported for certain statements on issues such as affirmative action, policing, gun rights, biological sex, and the COVID-19 vaccine, the boundaries of acceptable dialogue are becoming increasingly unclear [7]. This broader climate shapes expectations in medical education, where the question of how or whether to address such sensitive topics remains contested in the medical education literature [8,9].

In a prior study, two members of the current team explored medical educators’ approaches to politically charged topics using Hess’s framework (avoidance, denial, privilege, balance) and found that educators adopted varied stances: some declared their position to learners, while others took a balanced approach, focused on medical aspects, and withheld personal views [1]. However, educators remain divided on whether engaging with these charged topics cultivates critical thinking and communication skills or risks introducing bias and institutional endorsement of particular viewpoints [9,10]. Research on self-disclosure is complex: while general self-disclosure fosters rapport, political self-disclosure functions differently. Students who share an instructor’s views may feel affirmed, but those with differing perspectives often prefer that the faculty refrain from disclosing, as it can discourage the students’ participation [11,12]. There is a lack of literature on how students actively position themselves within these charged topics moments in medical education—whether as advocates, observers, or cautious participants. Understanding these patterns of student positioning is critical, as it influences both the quality of discussion and the distribution of emotional and intellectual labor in the classroom. However, avoidance of charged topics altogether risks reinforcing educational cultural hegemony – the implicit assumption that learning environments are culture and context-free, which can marginalize students’ perspectives and foster isolation [13]. Guided by this framework, which argues that the values of those in power become so embedded in education that they seem like neutral ‘common sense’ rather than one group’s perspective, this study explores medical students’ preferences related to the discussion of charged topics [13].

For the purpose of this research, “charged topics” are defined as topics that hold contradictions, creating both friction and potential in discussion [9]. The aim was not to probe political opinions but rather to examine students’ preferences and stances—whether passive, engaged, outspoken, or defensive- when encountering differing views. The research questions are: How do medical students position themselves during discussions of charged topics? How do medical students want medical educators to address and engage with them around charged topics?

## Methods

### Research Paradigm

This qualitative research study employed a critical constructivist approach, emphasizing that knowledge is socially constructed and shaped by power and ideology [14]. This paradigm aligns with our research question, which explores how medical students interpret and navigate charged conversations that are influenced by hierarchical and cultural power structures. The approach is also consistent with our conceptual framework of educational cultural hegemony, which examines how dominant ideologies shape what is considered normative or marginalized in educational contexts [13]. The interview questions were designed to elicit meaningful, rich, and thoughtful dialogue, followed by iterative review and discussion within the research team to develop a shared understanding of what participants said and what they left unsaid.

### Educational Setting and Participant Sampling

The study was conducted in 2024 at The George Washington School of Medicine and Health Sciences (GW), a private, federally chartered four-year medical program known for its diverse student population. The research team sent recruitment emails to medical students from all four years via class list-servs. Students completed a demographic form and were purposefully selected to ensure diversity reflective of the student population.

Sample size was guided by the concept of information power, which refers to the degree to which a sample contributes pertinent and comprehensive information [15]. Given the focused study aims, the research team estimated a total of twenty interviews, with approximately 4–5 participants from each class, to ensure a comprehensive understanding of the range of experiences across GW’s medical school. After each interview, the team evaluated the quality of data obtained, the range of perspectives included and whether additional data contributed new insights to the study’s themes. Institutional Review Board approval for human subject research was obtained prior to commencement (NCR235129).

### Data Collection

The research team created three scenarios based on their own teaching experiences and follow-up prompts related to the research questions (Table 1). The scenarios served to sensitize participants to discussion of charged topics in a classroom setting, including affirmative action, birth control and abortion coverage, and gun control (Supplemental Digital Appendix 1 for full interview guide). A trained research assistant (MM), not on faculty in the medical school and not known to the students, conducted all interviews virtually, with most lasting 60–90 minutes. With participant consent, interviews were recorded and transcribed using a secure online transcription service, reviewed for accuracy, and de-identified.

**Table 1:** Vignettes used to ask twenty U.S. medical students how they would like faculty members to respond to charged topics.


Scenario 1	During your weekly small group learning session, you overhear two fellow students talking. One says, “It seems that since the Supreme Court decision related to affirmative action, GW’s med school holistic admission criteria favor Black students. I think admission should be purely merit based.” You see that the session instructor overheard what that student said. What do you think should happen next?

Scenario 2	As part of a small group session on health policy and practice in the family medicine clerkship a faculty member is discussing insurance policies for various conditions. Your peers begin to discuss insurance coverage for birth control and abortion. One student asks for the faculty member’s viewpoint regarding whether insurance should cover birth control and abortion. What do you think should happen next?

Scenario 3	You’re in a small group session about gun violence. When a debate arises about whether guns should be more tightly regulated and whether regulation to gun access is insufficient, a student asks for literature about the value of gun control. What do you think should happen next?


### Data Analysis

Transcripts were uploaded to Dedoose® (version 9.2), an online platform for collaborative qualitative data analysis. We applied Braun and Clarke’s six-phase approach to reflexive thematic analysis, which involves familiarization with the data, coding, developing and reviewing themes, defining and naming them, and producing the final report [16]. Thematic analysis allowed for systematic coding while remaining flexible to incorporate both inductive insights and theoretically informed interpretations, consistent with our critical constructivist paradigm. Guided by the concept of educational cultural hegemony, the research team paid particular attention to instances where student perspectives were silenced, marginalized, or privileged within the learning environment.

Two research team members (MA and SS) independently reviewed the first three interviews conducted to generate codes. Subsequently, three research team members (MA, SS, and MM) independently coded the remaining interviews, with two members coding each one. Through iterative collaborative analysis, the research team developed and refined themes from initial codes, ensuring a rigorous and comprehensive interpretation that captured the nuanced experiences of participants.

### Critical Reflexivity

Reflexivity was employed to address potential bias in data interpretation. The research team comprises individuals with diverse social identities and professional roles: faculty and clinical educators with varying experiences facilitating discussions of charged topics, and researchers with expertise in qualitative methods and social justice. Team members engaged in explicit reflexive dialogue through virtual meetings and email exchanges to surface assumptions about neutrality and authority, reflect on how their own backgrounds and political beliefs might influence interpretation, and consider how their positionality informed analytic decisions. When interpretations diverged during coding and analysis, the team examined how their varied relationships to institutional power and commitments to equity shaped their perspectives. Importantly, the team maintained awareness that this study privileges the learner’s vantage point, and reflexive practice helped ensure analyses remained grounded in students’ experiences rather than the researchers’ own facilitation practices.

## Findings

Participants (N = 20) had a mean age of 27.7 years and represented all four years of medical school (Table 2). The sample was predominantly cisgender women (55%), with racial/ethnic diversity including Asian (30%), White (25%), Black/African American (20%), and Middle Eastern (10%), Hispanic/Latinx (5%), and multiracial (5%). One-third identified as LGBTQ+ (35%). Participants were from a range of US regions, and 15% were international.

**Table 2:** Sociodemographic characteristics of the twenty students interviewed May-July 2024.


SAMPLE CHARACTERISTICS	*N*	M

**Gender**		

Cis-Woman	11	

Cis-Man	8	

Non-binary	1	

**Graduation Year**		

2024	6	

2025	5	

2026	3	

2027	6	

**Sexual Orientation**		

Bisexual	2	

Gay or Lesbian/Queer	5	

Straight	13	

**Age**		27.7

**Home state/country of origin**		

US	17	

International	3	

**Race/ethnicity**		

Asian	6	

Black/African American	4	

Bi-racial	1	

Hispanic/Latinx	1	

Middle Eastern	2	

White/Caucasian	5	

Multi-ethnic	1	


The research team identified four themes on student engagement with charged topics, how students position themselves, and their preferences for educators’ engagement. Theme 1 – Constrained Voices: How hierarchy shapes student voice and expectations of educators describe how students’ engagement and positionality with charged topics are influenced by power hierarchies within the classrooms. In theme 2, Burdened Voices: Marginalization and unequal labor in charged discussions, students describe the undue burden on those from marginalized backgrounds to fill in the gaps for their peers and provide context and explanations for racial groups. Theme 3 Finding Voice: How students assert agency in charged discussions highlights how, despite hierarchical constraints, students describe using personal experience and voice to center patient perspectives and assert their opinions within the learning environment. Theme 4 Guiding Voice: Student expectations of educator engagement in charged discussions emphasize how students prefer educators to use their experiences and evidence-based knowledge in facilitating such discussions, and how neutrality and silence from an educator are viewed as opinions in themselves.

### Theme 1 Constrained Voices: How Hierarchy Shapes Student Voice and Expectations of Educators

Participants felt a need to discuss charged topics in educational spaces so they could prepare for conversations that might arise with patients or on public health topics. Yet, they described how power differences in educational spaces shaped what felt safe to say.

Participants brought up the need for “an opportunity to have a conversation” (P6) and highlighted the importance of discussing charged topics as part of the curriculum due to its direct relationship to patient care. However, the participants’ comfort in discussing such topics was tied to their perception of the educator’s authority and power, as well as concerns about what their peers might think of them.

The educator’s position of authority alone was enough to prevent students from speaking up, amplifying dominant narratives. “Students may not feel comfortable speaking their minds if a teacher … says that this is what they believe in, the students would need to obey that or to respect that, especially in medical settings.” (P18)

Some participants reported feeling unsafe and disheartened when they heard educators’ views that opposed their own. For example, P9 noted that “it might affect future interactions with and my perspectives on faculty, depending on what their views are.” Participants also feared potential consequences for discussing charged topics because educators were also their evaluators. “I’ll be afraid that I’d be affected in some way, whether they’re grading me or evaluating me. Maybe their viewpoint will affect how they view me.” (P1) Participants noted that the educator’s view of them was of particular importance in clinical years as the grading policies are “very subjective.” (P19) For students, the stakes are high if they do fail: “I don’t have a choice to fail here, so therefore I must keep my mouth shut. Otherwise, I face over $400,000 of debt.” (P10)

The importance of safe space and ground rules came up repeatedly as participants discussed the context for these conversations. The context was especially important in settings where hierarchy was diminished, such as in small groups. Weekly small groups, where close relationships with classmates and faculty are formed, provided such contexts, “…there are a bunch of data points that they’ve gathered about me, the person, that if I say something they don’t agree with, then also, we’ve joked and laughed and spent a hundred other sessions together.” (P13) In contrast, participants expressed hesitation in addressing charged topics in larger groups, such as clinical settings or lecture halls, where they felt less safe to speak up because of the number of people present.

### Theme 2 Burdened Voices: Marginalization and Unequal Labor in Charged Discussions

Factors such as religion, gender, race, and ethnicity influenced how participants interpreted comments in the learning environment. Participants who identified as Black or from underrepresented backgrounds acknowledged that some discussions place an undue burden on them to defend and educate their peers and faculty. “Things like (affirmative action favors Black people) have been said in my small group…and my instructor kind of just glosses over and doesn’t really address it in any way; and it leaves the onus on the students to voice their concerns. …A lot of times, it’s difficult, especially if the student is a Black student who’s underrepresented in medicine, to put themself in the center of that conversation and discuss it with their peers.” (P2)

Another participant reflected on the cumulative toll of such exchanges about affirmative action: “My identity as a Black woman in academia has been literally littered with comments [that] perpetuate things like imposter syndrome, and stereotype threat….” (P16) Despite this weight, many still felt compelled to speak up, sometimes because remaining silent carried its own form of moral injury.

The repetitive nature of some charged discussions while not listening or implicitly devaluing marginalized voices and experiences contributed to participants frustration and moral distress. One participant, who grew up interpreting for her immigrant parents in medical settings, explained during one small session why children “should not be interpreters” (P8), drawing on both personal experience and research. When a peer later repeated, “We should have the child do it,” she felt her contribution had been dismissed, highlighting the burden of repeatedly having to correct assumptions.

Educators from marginalized backgrounds were often better received when discussing charged topics, as their lived experiences provided authenticity and added depth to the conversation. One participant explained, “I think that I would be more willing to hear someone’s opinion (about affirmative action) … if they are a member of a marginalized community.” (P15)

### Theme 3 Finding Voice: How Students Assert Agency in Charged Discussions

Participants discussed how medical practice does not exist in a vacuum – healthcare intersects with social issues like access disparities, institutional policies related to minorities, and reproductive rights. Discussing charged topics was described as critical because avoiding such discussion could cause students to feel isolated and ultimately result in physicians who are ill-equipped to navigate the complex social realities their patients face.

Participants noted the “expectation that the faculty would address a comment, for example, pertaining to affirmative action favoring Black students” (P11) as opposed to just trying to table the discussion or saying, “Discuss it outside of class” (P2) or just “sweeping it under the rug.” (P1)

The aim of such discussions was seen to center patient care, preserving its quality and equity, rather than changing the mindset or opinions of peer learners. “I’m not here to convince John Smith in my class. I’m here to make sure his or her, their future patients are getting the best care possible, which would happen when we…. find a more truthful understanding of the world around us.” (P9) The participants noted that prioritizing dialogue about charged topics was crucial for deconstructing siloed thinking and checking biases that can impact clinical judgment, otherwise training doctors: “are going to come out of residency as biased individuals, and they’re going to provide care in a biased manner. Ultimately, biased care is subpar” (P4). The students valued diverse points of view, and learning from each other, but also understanding the context from their peers’ differing points of views and humanizing the conversations by associating “(patient)faces to these concepts.” (P8).

For many, speaking up was tied to supporting their peers or advocating for patients: “So much of being an ally is just showing up in those moments.” (P11) Participants often grounded their position in personal experience, for example around topics like gun control, “Our generation has lived through a lot of mass shootings, so I don’t think there’s any harm in saying my opinion, saying my own experiences with them, because unfortunately, we’ve had a lot.” (P8) Others expressed a sense of responsibility to stand up for patient’s right, for example in view of reproductive rights, noting that assumptions that “deny people autonomy” (P9) should not determine who receives care.

Some participants felt certain topics had a clear ethical and professional “right answer”, viewing keeping those topics as neutral as unnecessary or even frustrating. “Why is it being kept neutral? I don’t think there’s a reason to.” (P19) Another questioned the need for evidence, “I have this thing where it really sucks that people don’t believe things are true unless they see the study.” (P16)

Participants felt that being neutral as a student meant for some “[I] don’t know if I know enough about a topic to even say one way or another” (P10) while others were concerned that expressing a perspective might hurt a peer’s feelings or worse, lead to negative repercussions.

### Theme 4 Guiding Voice: Student Expectations of Educator Engagement in Charged Discussions

Participants described ideal educators as those who guide student learning objectively and professionally while sharing their experiences and perspectives. They also expected educators to be knowledgeable, able to discuss relevant facts and address unexpected topics- such as affirmative action- confidently.

Students particularly valued educators sharing evidence-based knowledge grounded in guidelines and clinical experience, emphasizing practical, real-world insights and their relevance to clinical practice.“I like to usually stick to more concrete and objective facts or characteristics about a person. […] it’s more about their expertise and their educational background,” (P18) and “it’s also important to hear from, again, disproportionately emergency medicine physicians who are dealing with the wounds where those bullets go… from an expert, someone with experience’s standpoint.” (P13)

Participants expressed a curiosity, if not a clear desire, to know educators’ perspectives on the scenarios and valued it when educators openly shared their views. One reason for appreciating this openness was that it helped students learn and formulate their own opinions. “Especially this person being a mentor and someone who we respect, I would want to also hear their opinion, because [how] they’re thinking about it could impact my thinking, but also, of course, could impact my perception of them.” (P16) Another reason was that it allowed them to assess whether they could trust the educator to be non-discriminatory and not harbor racist views. Additionally, attempts by educators to remain neutral were often seen as problematic: “If they are not disclosing their opinion, and then if the faculty happens to talk on the subject later on and gives evidence for or against, then it feels as if they’re covertly trying to influence the opinion of the crowd that they’re instructing, which would dissuade me as an individual from trusting them.” (P10)

Educators’ silence was seen as a form of power as well as an opinion, acting as a form of hegemonic reinforcement: “I think it would be very telling of them to not say anything.” (P17) Or if they are not openly sharing their perspective, they are hiding something “you get this sense that the individual is hiding whatever it is that they actually feel.” (P10)

Several participants suggested a bi-directional approach where educators could facilitate discussion first, allow students to exchange viewpoints, and only then share their own, an approach described as: “Let’s have a discussion about both sides… and then… I’d be more than happy to share my thoughts.” (P4)

## Discussion

The team’s findings revealed complex perspectives among medical students regarding their own personal engagement with charged topics and their preferences for educators’ engagement in the learning environment. Participants expressed hesitancy to speak up in hierarchical learning environments. Those from marginalized backgrounds emphasized the importance of safety in these learning spaces, which they characterized as non-hierarchical environments, free from judgment and personal or professional repercussions. Discussions were enhanced when sharing of perspectives was bi-directional, and educators drew upon evidence-based guidelines and their own clinical experience, while maintaining a focus on patient care outcomes and social responsibility.

Students’ desire for a learning environment where there is a range of ideas and independent thought echoes principles of democratic education where students are empowered to learn through dialogue, valuing diverse perspectives and the deeper understanding of concepts [17,18]. While dialogical education focuses on learning through dialogue, democratic education focuses on the dynamic of power and oppression in society and in the classroom [17]. However, each is a continuum of the other, and both focus on student agency, equity, and reduction of hierarchy in the classroom [19]. Hess notes that democratic classrooms are less individualistic and oriented towards the common good where the ideological diversity found amongst students within a classroom provides for teaching tolerance on varying perspectives [20]. The democratic classroom is especially important when educating future doctors who will be treating patients with different sets of values and opinions. Framing the importance of talking across differences through patient-centered care brings to the forefront that the study’s participants wanted patient care to be in the heart of such discussions.

Participants stressed the importance of safety in learning spaces. This safety enabled open discussion of charged topics as they arose. This concept aligns with the broader educational literature showing that addressing uncomfortable but important conversations helps students feel seen in their backgrounds, experiences, and feelings. Such discussions also improve subsequent engagement with classroom material [21,22].

Students in this study felt uncomfortable with the silent instructor; this silence was taken as an expressed opinion, or what has been described as a negative acknowledgement. Educators’ silence can reinforce an oppressive, dominant view over those of the marginalized view and lived experience [23,24]. Similar sentiment occurred among certain students when educators stayed neutral in discussions that directly affected them, like reproductive rights and affirmative action.

Neutrality can provide a foundation for unbiased and fair discussions; however, it is not always possible or desirable in the context of democratic education, as classrooms are inherently not neutral [20]. Students often voiced their desire to know educators’ opinions; but, this is a double-edged sword. Recent surveys show that about 64% of higher education faculty occasionally to often feel they can’t express their opinions due to fear of how administrators, other faculty, or students might react, while one in five have self-censored in some way in a professional context [5]. While some students maintained neutrality in the classroom as they were still forming their opinions or avoiding hurting their peers, they did not extend the same explanation to an educator’s neutrality. Educators are often expected to be the main knowledge-bearer, part of the “college professor” stereotype carried around by both educators and students of what their roles should be [20,23,25].

### Implications for Practice

**Figure 1:**
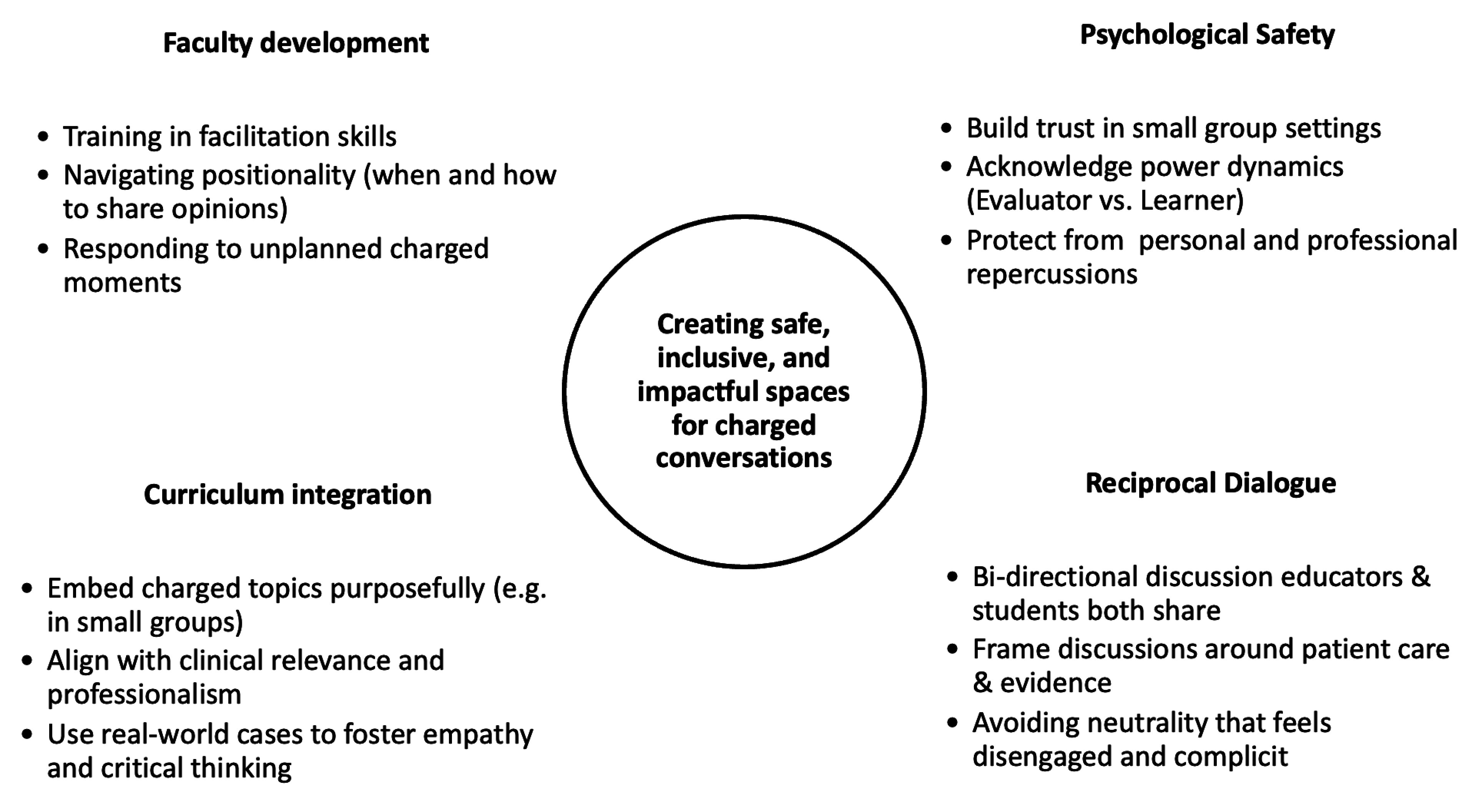
Recommendations for addressing charged topics in medical education.

Faculty development in facilitation of charged discussions is crucial to prevent marginalizing students with alternate points of view and to ensure safe spaces. The students leaned towards authenticity and appreciating knowing their educator’s point of view, even if it contradicted their own. However, there is a fine line between authenticity and creating psychologically safe spaces that would require not just faculty development but also deep reflection and potentially years of experience in dealing with charged conversations, especially those that arise unplanned in the classroom. Hess and Kite propose that a facilitator’s positionality can be used as a pedagogical tool, with the caveat of explaining to students the purpose of sharing their positionality. Such sharing requires bringing opinions and identities to the foreground in some instances or choosing to withhold an opinion in others to promote a discussion in the classroom [20,23]. Most importantly, purposefully and thoughtfully incorporating charged topics into the longitudinal small group curriculum can help in reinforcing effective spaces for such discussions. While students mainly emphasized the role of the educator in guiding charged discussions, medical educators will also need to prepare students to engage in these conversations effectively. A summary of our study findings and implications is found in Figure 1.

### Limitations

The geographical and institutional context of our investigation—a single academic center situated on the East Coast of the US- potentially reflects predominant progressive ideological orientations that may exhibit homogeneity on certain topics. Nevertheless, the findings presented can inform other institutions’ pedagogical approaches to evaluating their specific contextual environments and engaging relevant stakeholders in deliberations regarding the integration of charged topics within curricular frameworks. Although the sensitizing scenarios employed in this inquiry are predominantly contextualized within the US sociopolitical landscape, potentially limiting international generalizability, the global phenomena of transnational population movement and increasing incidence of firearm-related mortality and morbidity across multiple geographical regions suggest that these findings may catalyze meaningful discourse beyond US territorial boundaries.

## Conclusion

The team’s findings shed light on the nuanced views of students regarding engagement with charged topics and their preferences about educators’ facilitation of such topics. These insights are crucial for grasping students’ expectations of educators amidst today’s diverse and politically polarized environment. Additionally, the findings suggest that students expect medical educators to be authentic while sharing perspectives but also maintaining a safe space for discussing charged topics. While educators cannot completely ensure that spaces are safe for all learners, if they are provided with faculty development to undertake charged conversations, they could prepare students for the complex social and political landscapes the students will encounter in clinical practice.

## Additional File

The additional file for this article can be found as follows:

10.5334/pme.2253.s1 Appendix 1. Interview Guide.
