# Supplementary material for: Charged Topics in Medical Education: Students’ Perspectives on Power, Voices, and Faculty Engagement
Source: Perspect Med Educ. 2026 Feb 3;15(1):65–74. doi: 10.5334/pme.2253 (PMC12880022; doi:10.5334/pme.2253)
Supplement: Appendix 1. — Interview Guide. [file pme-15-1-2253-s1.pdf]

Interview Guide

(This is a preliminary guide, which may undergo changes after pilot-testing)

Interview duration: 45 minutes

Introduction:

I am XYZ. This interview is part of a research study which aims to understand approaches students take in situations where they encounter politically charged topics in academic settings and how they prefer medical educators to engage with these politically charged topics in the curriculum.

Opening statement:

During this interview we will explore how individuals deal with politically charged topics in the educational context. (Read out or provide the interviewee with the scenarios)

Scenarios #1

During your weekly small group learning session, you overhear two fellow students talking. One says, "It seems that since the Supreme Court decision related to affirmative action (institution)'s med school holistic admission criteria favors Black students. I think admission should be purely merit based." You see that the session instructor has also overhead what that student said.

What do you think should happen next?

[Interviewer can provide the following examples if needed, but does not have to read them all out:]

a. Instructor speaks to the group and discloses that they support (institutions)'s position on affirmative action.
b. The instructor says nothing.
c. The instructor comments that groups will often have different perspectives on such topics, and it is important to respect all points of view.
d. The instructor stops the class and talks about the recent supreme court decision striking down affirmative action and shares their own position on the topic.
e. The instructor speaks to the student separately after class and state that the comment could be viewed as offensive by others.
f. The instructor reports the student and the comment to the Deans office.
g. Other way you think the instructor should handle the comment?

If you were in this group, would you want to know your instructor's position on the topic? If yes, why? Would you still want to know even if their position was the opposite of yours? Are there are any instructor characteristics that might influence your answer

In such a situation would you state your own position about the topic to the rest of the group? If yes (or no), why?

If this topic was opened up to discussion in class, how would you feel (and deal with) if someone in class had opposing point of view on this topic? How would you approach the discussion?

What would be a satisfying end point to this discussion?

Scenarios #2

As part of a small group session on health policy and practice in the family medicine clerkship a faculty member is discussing insurance policies for various conditions. Your peers begin to discuss insurance coverage for birth control and abortion.
One student asks for the faculty member's viewpoint regarding whether insurance should cover birth control and abortion.

What do you think should happen next?

[Interviewer can provide the following examples if needed, but you do not have to read them all out:]

a. They do not disclose their position (pro-life or pro-choice).
b. The instructor shares their position on the topic as a physician.
c. The instructor discusses how their position as a physician and as a parent / Catholic (or any other identities) can sometimes conflict on issues.
d. They discuss the recent supreme court decision and its implications
e. The instructor advises the student to have this discussion outside the classroom.
f. Other ways you think the instructor should handle the comment?

In such a situation would you state your own position about the topic to the rest of the group? If yes (or no), why?

If this topic was opened up to discussion in your classroom, how would you feel (and deal with) if someone in class had opposing point of view on this topic? How would you approach the discussion?
What would be a satisfying end point to this discussion?

Scenario #3:
You're in a small group session about gun violence when there is a debate that guns should be more tightly regulated and that regulation to gun access is insufficient, another student asks for literature about the value of gun control. What do you think should happen next?

[Interviewer can provide the following examples if needed, but you do not have to read them all out:]

a. The instructor to disclose their personal position on gun control.
b. Instructor asks students to look for healthcare related evidence for and against gun control.
c. The instructor comments that groups will often have different perspectives on such topics and it is important to respect all points of view.
d. What would be a satisfying end point to this discussion?

If you were in this group, would you want to know your instructor's position on the topic? If yes, why? Would you still want to know even if their position was the opposite of yours? Are there are any instructor characteristics that might influence your answer

In such a situation would you state your own position about the topic to the rest of the group? If yes (or no), why?

Additional Probes:
When you come across a scenario like the samples we provided, what do you tend to do and how do you react? (Additional probe (AP) # A,B,C,D)
<u>AP:</u>

A. Looking at the scenarios provided do you recognize situations/discussions that you have personally encountered? Can you tell more about what happened in these situations / discussions? How you felt and what you would have liked the instructor to do differently?
B. What is your view about the role of teachers when these types of situations are encountered? Do you believe their role is to keep the discussion neutral? How comfortable do you feel it is to voice your personal stance on the issue at hand?
C. Share some personal experiences you might have had dealing with controversial topics in the educational setting. What happened and how did you (or others deal with the situation?
    a. Has the instructor/lecturer have an opposing opinion? How did that make you feel?
D. Can you think of an instance/example where you felt that such a discussion could have been used as a learning moment for participants and yourself to generate a broader understanding about such sensitive issues?
